# The impact of lifestyle on adherence to treatment in a sample of patients with Major Depression

**DOI:** 10.1192/j.eurpsy.2023.756

**Published:** 2023-07-19

**Authors:** D. Conti, N. Girone, S. Vanzetto, M. Cocchi, F. Achilli, S. Leo, M. Bosi, B. Benatti, B. Dell’Osso

**Affiliations:** 1 Department of Mental Health, Department of Biomedical and Clinical Sciences “Luigi Sacco”; 2 Department of Health Sciences, Aldo Ravelli” Center for Neurotechnology and Brain Therapeutic, University of Milan, Milan, Italy; 3 Department of Psychiatry and Behavioral Sciences, Bipolar Disorders Clinic, Stanford University, Stanford, CA, United States; 4 “Centro per lo studio dei meccanismi molecolari alla base delle patologie neuro-psico-geriatriche”, University of Milan, Milan, Italy

## Abstract

**Introduction:**

Poor adherence to treatment is currently stated to be one of the causes of depression relapse and recurrence.

**Objectives:**

Aim of the present study was to assess potential differences in terms of clinical and socio-demographic characteristics specifically related to adherence to treatment features, medical comorbidities, and substance abuse in a sample of patients diagnosed with Major Depression in an Italian psychiatric department.

**Methods:**

Patients with a DSM-5 diagnosis of Unipolar or Bipolar Major Depressive Episode, of either gender or any age were recruited from the Psychiatry Department of Luigi Sacco University Hospital in Milan. Main clinical and socio-demographic variables were collected reviewing patients’ medical records. Moreover, adherence to psychopharmacological treatment was assessed using the Clinician Rating Scale (CRS; Kemp et al, 1996; 1998). Adherence was defined as ratings of > or =5 on the CRS. Descriptive and association analyzes were performed, setting the significance level at p<.05.

**Results:**

80 patients with a diagnosis of Unipolar Major depressive episode (48.9%) and Bipolar Major Depressive Episode (51.1%) were included. For the purposes of the study, the total sample was divided into two subgroups based on adherence to pharmacological treatment (A+ vs A-). Significantly higher rates of inpatients from psychiatric ward were A- compared to A+ patients (84.6% vs 48.1%, p=.011). A- patients were significantly more unemployed (57.9% vs 23.8%, p=.015), were mostly living in their family of origin (50% vs 21.4%, p=.027), and had fewer years of education compared to A+ subgroup (10.52±3.28 vs 12.2±3.1 years, p=.053). Higher rates of Bipolar Depression diagnosis and a prevalent manic polarity lifetime emerged in A- compared to the A+ group (73.1% vs 42.3%, p=.010; 30.8% vs 3%, p=.011, respectively). Moreover, A+ reported significantly higher rates of depressive prevalent polarity lifetime (72.7% vs 30.8%, p=.011). A- reported significantly higher rates of comorbidity with alcohol or other substance use disorders lifetime (46.2% vs 5.7%, p=.006) and almost one involuntary commitment lifetime (23.1% vs 11.1%, p=.013).

**Conclusions:**

In our sample adherence to treatments showed significant differences in terms of clinical and socio-demographic characteristics. Low levels of adherence have been associated with higher hospitalization rates, involuntary commitments, greater comorbidity with alcohol or drugs. Our data therefore seem to suggest that less adherence leads to a worse disease course and a worse quality of life. It therefore appears useful to include an assessment of adherence in the clinical practice and implement interventions to improve therapeutic adherence and ensure a better quality of life.

**Disclosure of Interest:**

None Declared

